# Clinical outcomes of de novo metastatic HER2-positive inflammatory breast cancer

**DOI:** 10.1038/s41523-023-00555-w

**Published:** 2023-06-02

**Authors:** Ana C. Garrido-Castro, Meredith M. Regan, Samuel M. Niman, Faina Nakhlis, Claire Remolano, Jennifer M. Rosenbluth, Caroline Block, Laura E. Warren, Jennifer R. Bellon, Eren Yeh, Beth T. Harrison, Elizabeth Troll, Nancy U. Lin, Sara M. Tolaney, Beth Overmoyer, Filipa Lynce

**Affiliations:** 1 Department of Medical Oncology, Dana-Farber Cancer Institute, Boston, MA USA; 2 Breast Oncology Program, Dana-Farber Brigham Cancer Center, Boston, MA USA; 3 Harvard Medical School, Boston, MA USA; 4 Department of Data Science, Dana-Farber Cancer Institute, Boston, MA USA; 5 Division of Breast Surgery, Department of Surgery, Brigham and Women’s Hospital, Boston, MA USA; 6 Department of Radiation Oncology, Dana-Farber Cancer Institute, Boston, MA USA; 7 Department of Radiology, Brigham and Women’s Hospital, Boston, MA USA; 8 Department of Pathology, Brigham and Women’s Hospital, Boston, MA USA; 9 Present Address: Helen Diller Family Comprehensive Cancer Center, University of California San Francisco, San Francisco, CA USA

**Keywords:** Breast cancer, Breast cancer, Metastasis

## Abstract

Inflammatory breast cancer (IBC) is a rare, aggressive form of breast cancer that presents as de novo metastatic disease in 20–30% of cases, with one-third of cases demonstrating HER2-positivity. There has been limited investigation into locoregional therapy utilization following HER2-directed systemic therapy for these patients, and their locoregional progression or recurrence (LRPR) and survival outcomes. Patients with de novo HER2-positive metastatic IBC (mIBC) were identified from an IRB-approved IBC registry at Dana-Farber Cancer Institute. Clinical, pathology, and treatment data were abstracted. Rates of LRPR, progression-free survival (PFS), overall survival (OS), and pathologic complete response (pCR) were determined. Seventy-eight patients diagnosed between 1998 and 2019 were identified. First-line systemic therapy comprised chemotherapy for most patients (97.4%) and HER2-directed therapy for all patients (trastuzumab [47.4%]; trastuzumab+pertuzumab [51.3%]; or trastuzumab emtansine [1.3%]). At a median follow-up of 2.7 years, the median PFS was 1.0 year, and the median OS was 4.6 years. The 1- and 2-year cumulative incidence of LRPR was 20.7% and 29.0%, respectively. Mastectomy was performed after systemic therapy in 41/78 patients (52.6%); 10 had a pCR (24.4%) and all were alive at last follow-up (1.3–8.9 years after surgery). Among 56 patients who were alive and LRPR-free at one year, 10 developed LRPR (surgery group = 1; no-surgery group = 9). In conclusion, patients with de novo HER2-positive mIBC who undergo surgery have favorable outcomes. More than half of patients received systemic and local therapy with good locoregional control and prolonged survival, suggesting a potential role for local therapy.

## Introduction

Inflammatory breast cancer (IBC) is a rare, aggressive form of breast cancer with poorer overall survival (OS) outcomes than non-IBC^1–4^. Among patients with IBC, 20–30% present with de novo metastatic disease (mIBC)^5–7^, as opposed to only 6–10% of non-IBC patients who present with de novo metastatic disease^8,9^. Approximately 35% of patients with de novo mIBC have HER2-positive (HER2+) disease, which presents opportunities for treatment with HER2-directed agents^8^. However, there are limited data regarding the efficacy of first-line HER2-directed therapies in de novo mIBC.

The current standard treatment for stage III IBC consists of tri-modality therapy, which includes neoadjuvant systemic therapy followed by modified radical mastectomy (MRM) and post-mastectomy radiation therapy^9^. The use of tri-modality therapy has been associated with 5-year OS rates ranging from 44% in patients with triple-negative IBC to 74% in patients with HER2 + IBC^10^. The incorporation of HER2-directed agents into tri-modality therapy has significantly improved survival outcomes for patients with HER2 + IBC^11^. In addition, experiencing a pathologic complete response (pCR) with neoadjuvant systemic therapy is a strong predictor of better OS^12^.

Patients with de novo mIBC are treated with systemic therapy upfront with consideration for local therapy (usually surgery and radiation), with the goal of providing improved locoregional control. However, the impact of locoregional therapy on OS in mIBC patients remains uncertain^13,14^. Importantly, patients with HER2+ mIBC are more likely to experience a durable distant disease response to induction systemic therapy than those with HER2-negative mIBC^15^. Therefore, patients with HER2+ mIBC may be potentially spared a greater risk of future morbidity associated with untreated locoregional disease should they receive local therapy. To explore this further, we conducted a retrospective study to evaluate patterns of systemic and locoregional therapy, locoregional progression or recurrence (LRPR), and survival among patients with de novo HER2+ mIBC treated at a single institution.

## Results

### Patient characteristics and treatment

A total of 78 patients met the inclusion criteria and were analyzed in this study. Patient and disease characteristics at mIBC diagnosis are included in Supplementary Table 1. The median age at diagnosis was 53 years (range 24–91 years). Sites of metastatic disease at presentation included visceral (*n* = 40; 51.3%), bone and/or contralateral or distant lymph nodes (*n* = 34; 43.6%), and central nervous system (CNS) with extracranial disease (*n* = 4; 5.1%). Hormone receptors, i.e., estrogen and/or progesterone receptors, were positive (≥10%) in 41 (52.6%) patients. Most patients had either one (*n* = 42; 53.8%) or two (*n* = 23; 29.5%) metastatic sites at presentation. Initial HER2-directed therapy included trastuzumab (*n* = 37; 47.4%), trastuzumab plus pertuzumab (*n* = 40; 51.3%), or trastuzumab emtansine (T-DM1; *n* = 1; 1.3%). Details of the chemotherapy backbone used with the initial HER2-directed therapy are included in Supplementary Table 1. Most patients (*n* = 65; 83.4%) received taxane-based chemotherapy along with initial HER2-directed therapy.

In 41 patients (52.6%), mastectomy was performed for the primary tumor after receipt of systemic therapy (Supplementary Table 2), with the majority undergoing surgery (85.4%) within 12 months of mIBC diagnosis. Most patients who underwent surgery had a mastectomy and axillary lymph node dissection (*n* = 36; 87.8%). Radiation therapy for locoregional disease was administered in 33/41 patients (80.5%), 3 of whom received radiation prior to surgery (7.3%).

### Survival

**Fig. 1:**
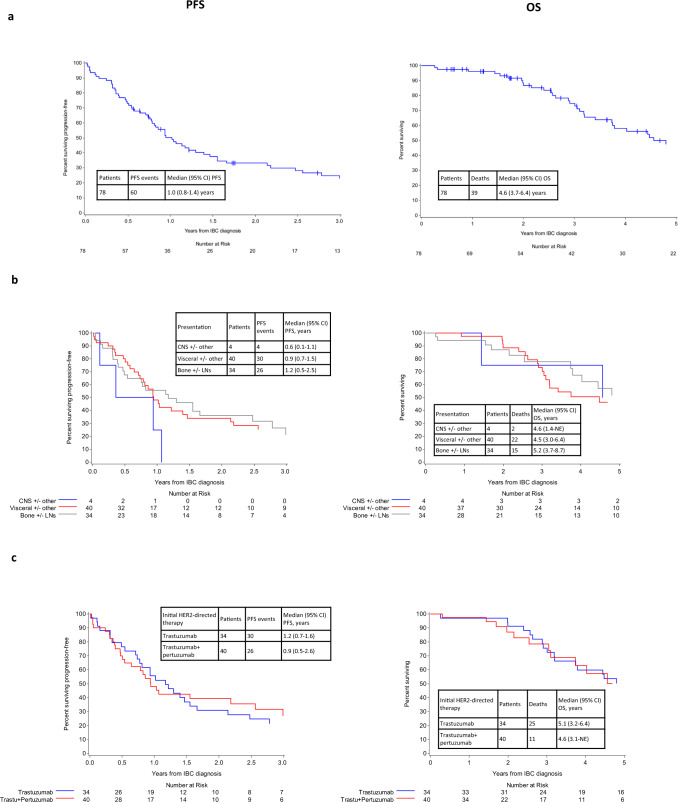
Progression-free survival (PFS) and overall survival (OS) from diagnosis in patients with de novo metastatic HER2-positive inflammatory breast cancer (IBC). Median follow-up in overall cohort: 2.7 years (yrs). **a** Overall cohort. **b** According to sites of disease at presentation (CNS ± other [blue color]; visceral ± other [red color]; bone ± LNs [grey color]. **c** According to initial HER2-directed therapy (trastuzumab [blue color]; trastuzumab+pertuzumab [red color]). CI confidence interval, CNS central nervous system, HER2 human epidermal growth factor receptor 2, LN lymph node, NE not estimable, trastu trastuzumab.

After a median follow-up of 2.7 years, 60 patients (76.9%) had experienced progression-free survival (PFS) events and 39 patients (50%) had died. The median PFS was 1.0 year (95% confidence interval [CI]: 0.8–1.4), and the median OS was 4.6 years (95% CI: 3.7–6.4) from mIBC diagnosis (Fig. 1a). Among the 34 patients with only contralateral axillary or distant lymph node involvement and bone metastases, the median PFS and OS were 1.2 years (95% CI: 0.5–2.5) and 5.2 years (95% CI: 3.7–8.7), respectively. In patients with visceral metastases (*n* = 40), the median PFS and OS were slightly shorter of 0.9 years (95% CI: 0.7–1.5) and 4.5 years (95% CI: 3.1–6.4), respectively (Fig. 1b).

Among the 37 patients who were treated with trastuzumab as initial HER2-directed therapy, the median PFS was 1.2 years (95% CI: 0.7–1.6) and the median OS was 5.1 years (95% CI: 3.2–6.4). Among the 40 patients who were treated with trastuzumab and pertuzumab, the median PFS was 0.9 years (95% CI: 0.5–2.6) and the median OS was 4.6 years (95% CI: 3.1-not estimable) (Fig. 1c). Among the patients without known CNS metastasis at mIBC diagnosis (*n* = 74; 94.9%), the cumulative incidence of CNS metastasis was 15.8% (95% CI: 8.3–25.4%) at 1 year and 23.8% (95% CI: 14.3–34.7%) at 2 years (Supplementary Fig. 1).

**Fig. 2:**
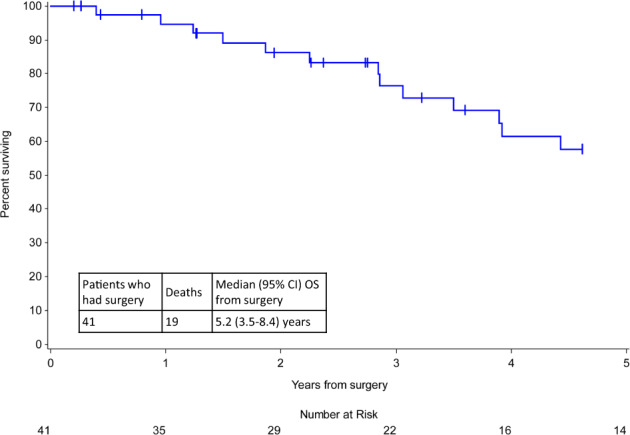
Overall survival (OS) from the time of surgery in patients with de novo HER2-positive metastatic inflammatory breast cancer (mIBC) who underwent mastectomy. Among patients diagnosed with de novo HER2-positive mIBC who underwent mastectomy (*n* = 41), the median OS from surgery was 5.2 years (95% CI: 3.5–8.4). CI confidence interval, HER2 human epidermal growth factor receptor 2.

For patients who underwent surgery, the median OS from the date of surgery was 5.2 years (95% CI: 3.5–8.4 years) (Fig. 2). pCR occurred in 10/41 patients (24.4%), all of whom were alive at 1.3–8.9 years after surgery (Supplementary Table 2) (Fig. 2).

### Locoregional progression or recurrence

**Fig. 3:**
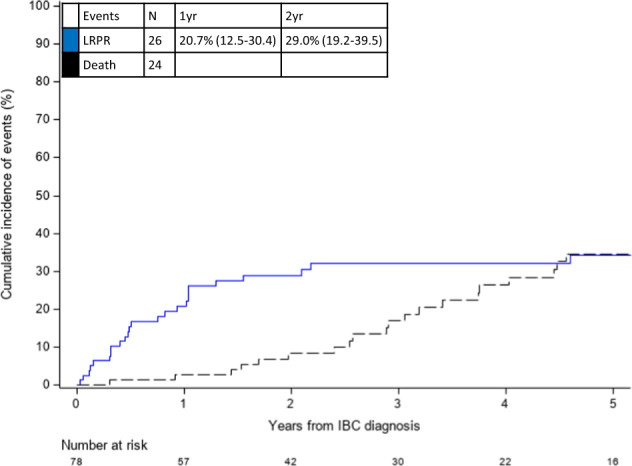
Cumulative incidence of locoregional progression or recurrence (LRPR), with competing risk of death, among patients with de novo HER2-positive metastatic inflammatory breast cancer (mIBC). Among patients diagnosed with de novo HER2-positive mIBC (*n* = 78), the cumulative incidence of LRPR was 20.7% (95% CI: 12.5–30.4) and 29.0% (95% CI: 19.2–39.5) at 1 and 2 years, respectively. Cumulative incidence of LRPR is shown as the blue line; competing risk of death is shown as the dashed black line. CI confidence interval; HER2 human epidermal growth factor receptor 2; yr year.

Among the overall cohort, LRPR occurred in 26/78 (33.3%) patients. The cumulative incidence of LRPR was 20.7% (95% CI: 12.5–30.4%) at 1 year and 29.0% (95% CI: 19.2–39.5%) at 2 years from mIBC diagnosis (Fig. 3).

**Fig. 4:**
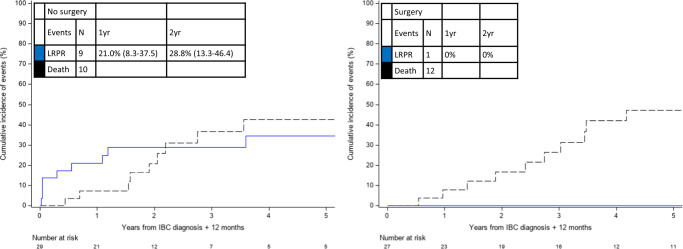
Cumulative incidence of locoregional progression or recurrence (LRPR), with competing risk of death, among patients with de novo HER2-positive metastatic inflammatory breast cancer (mIBC) who were alive and free of LRPR at 12 months since diagnosis, according to receipt of surgery. Among patients diagnosed with de novo HER2-positive mIBC alive and free of LRPR at 12 months from diagnosis (*n* = 56), 10 patients subsequently developed LRPR. The cumulative incidence of LRPR over the subsequent 1 and 2 years from the 12-month landmark was 10.8% (95% CI: 4.3–20.6) and 14.8% (95% CI: 6.9–25.7), respectively. Cumulative incidence of LRPR is shown as the blue line; competing risk of death is shown as the dashed black line. CI confidence interval; HER2 human epidermal growth factor receptor 2; yr year.

To investigate the association between the receipt of locoregional therapy and the risk of LRPR, a landmark analysis was performed in the subset of patients alive and free of LRPR at 12 months from mIBC diagnosis (*n* = 56/78, 71.8%). Among these patients, 27/56 (48.2%) had surgery within 12 months of diagnosis and 29/56 (51.8%) did not have surgery within 12 months of diagnosis. A total of 10 patients had experienced LRPR after the 12-month landmark, with cumulative incidence of LRPR over the subsequent 1 and 2 years from the 12-month landmark of 10.8% (95% CI: 4.3–20.6%) and 14.8% (95% CI: 6.9–25.7%), respectively (Fig. 4). Nine of those who have experienced LRPR had not undergone surgery. The one patient who had undergone surgery experienced an LRPR event at 7.8 years after the 12-month landmark. Thus, among patients who underwent surgery, the cumulative freedom from LRPR was 100% at 2 years after the 12-month landmark. The cumulative LRPR incidence was 21 and 29% at 1 and 2 years after the 12-month landmark, respectively, in patients who did not undergo surgery.

## Discussion

In the present retrospective study, 41 out of 78 patients with de novo HER2+ mIBC (52.6%) underwent mastectomy, with 33/41 (80.5%) also receiving radiation therapy. The median OS from the date of surgery was 5.2 years. Patients who underwent surgery experienced good locoregional disease control, with only 1 LRPR reported 7.8 years after surgery, and a 2-year LRPR rate of 0% in our landmark analysis of patients who had not experienced LRPR by 12 months after diagnosis of de novo mIBC. All patients who had a pCR (*n* = 10/41) were alive at the date of last follow-up (1.3–8.9 years from the date of surgery).

Several prior retrospective studies have reported lower LRPR occurrence rates and better survival outcomes among mIBC patients who underwent locoregional therapy. These studies were mostly single institution, not limited to de novo mIBC or to a specific cancer subtype^16,17^. In some of these studies, partial or complete response of distant disease to chemotherapy, receipt of MRM, or locoregional therapy were independently associated with improved OS on multivariate analysis^16,18^. In a large retrospective study that included 580 patients with de novo mIBC from the Netherlands Cancer Registry, the researchers performed propensity score matching, which resulted in a matched cohort of 101 surgery and 101 non-surgery mIBC patients based on variables including year of diagnosis, age, breast cancer subtype, use of targeted therapy, use of radiation therapy, and localization of metastases. In the matched cohort, the median OS was 22.4 months for patients who underwent surgery and 16.3 months for patients who did not undergo surgery (*p* < 0.005). Multivariable analysis of the matched cohort revealed that surgery was associated with longer survival (hazard ratio: 0.62; 95% CI: 0.26–0.79)^19^.

These retrospective studies^16–19^ must be interpreted with caution. The definition of de novo metastatic IBC was not clearly expressed in some studies, or it allowed patients who had identification of distant metastases within three months of the diagnosis of the primary tumor. Most importantly, in three of the studies^16–18^, OS was calculated from the date of diagnosis, which introduces guarantee-time bias to the OS estimates for patients who undergo surgery^20^. To correct this, van Uden et al.^19^ conducted a landmark analysis that only included patients who survived the first six months after diagnosis, with the reasoning that almost all patients would have had surgery within this time frame. To overcome this potential bias in our present analysis, survival was estimated from the date of surgery for the 41 patients who underwent surgery of the primary tumor.

To date, there have been no randomized controlled trials to study the survival impact of locoregional therapy in de novo mIBC. This is mostly due to the rarity of this breast cancer type, which introduces difficulty in conducting interventional trials powered to study OS. Thus, treatment guidelines for IBC patients have primarily been derived from randomized clinical trials of patients with non-IBC^13,14^. In non-inflammatory metastatic breast cancer, three randomized trials have produced conflicting results about the impact of locoregional therapy on survival^21–23^. While the phase III MF07-1 trial showed a 5-year OS of 41.6% in the upfront surgery group versus 24.4% in the systemic therapy-only group (*p* = 0.005)^21^, two other trials showed discordant results. A trial from Tata Memorial Center in Mumbai^23^ and a U.S. cooperative trial (ECOG-ACRIN E2108)^22^ revealed no improvement in OS with the addition of locoregional therapy of the primary tumor. Based on these results, locoregional therapy is not routinely offered to patients with de novo metastatic non-IBC.

Despite the lack of impact of primary tumor-directed locoregional therapy on OS in the E2108 study, the LRPR rate was significantly higher in the optimal systemic therapy (OST) arm (25.6%) compared with the OST + locoregional treatment arm (10.2%) (Gray test *p* = 0.003)^22^. For patients with metastatic IBC, nearly 60% experience LRPR if treated with systemic therapy alone^24^. Locoregional disease in breast cancer patients may significantly affect the quality of life and can lead to considerable pain, uncontrolled bleeding, and recurrent infections. Therefore, based on the accumulating body of evidence summarized here, it seems reasonable to consider local therapy for patients with de novo mIBC with controlled distant disease who are likely to do well without chemotherapy for the duration of surgery and radiation therapy.

In our retrospective study of patients presenting with HER2+ de novo mIBC, the median OS was 4.6 years. Lambertini et al.^25^ reported a comparison of outcomes in HER2-positive recurrent vs. de novo metastatic breast cancer, with a median OS of 4.7 years in the de novo group, which is numerically similar to the median OS observed in our cohort. Like our cohort, most patients received first-line trastuzumab-based therapy. Many publications to date that include outcomes of patients with de novo HER2+ metastatic breast cancer have not specified whether patients presented with IBC vs. non-IBC^26,27^. Overall, our data confirm that with receipt of HER2-targeted therapy, HER2+ mIBC has similar outcomes to HER2+ metastatic non-IBC, which is different from what has been reported with other subtypes of IBC, commonly associated with worse outcomes compared to non-IBC^16,18,19,28^.

Our study has several strengths. We used a well-maintained database of patients diagnosed with IBC by physicians with expertise in this disease. We also used a rigorous statistical analysis with a landmark analysis method to try to overcome some of the inherent biases associated with a retrospective study examining the relation of local therapy decision-making with OS. Nevertheless, this study was still vulnerable to selection bias related to receipt of locoregional therapy, since treatment recommendations were made at the discretion of the treating clinical team. As such, and in contrast to a randomized controlled trial, the difference in LRPR rate between the surgery and no-surgery group in the landmark analysis cannot be attributed solely to receipt of locoregional therapy.

IBC is a unique disease process with distinct clinical features differentiating it from non-IBC. Even in the absence of an OS benefit, locoregional therapy in addition to systemic therapy may be appropriate for a subset of patients, with the intent of avoiding the morbidity associated with LRPR in IBC. However, such an approach needs to take into consideration the potential risks associated with MRM and post-mastectomy radiation therapy, and balance against the competing risks of high rates of distant metastases. Thus, as advances in targeted therapies continue to improve survival outcomes for patients with metastatic IBC, additional studies are needed to elucidate the impact of tri-modality therapy on clinical outcomes and quality of life in this patient population.

## Methods

### Patient population

Patients diagnosed with de novo HER2+ mIBC between 1998–2019 were identified from an IRB-approved IBC registry at Dana-Farber Cancer Institute (DFCI). All patients were seen at a dedicated IBC program, where detailed information on patient and tumor characteristics, disease presentation and treatment for each patient was recorded. Eligible patients presented with signs and symptoms consistent with a clinical diagnosis of IBC, usually characterized by a rapid onset of diffuse erythema and edema (or peau d’orange) involving at least one-third of the skin, with or without an underlying palpable mass, as defined by the American Joint Committee on Cancer (8th edition)^29^. To be eligible for inclusion in this study, patients were required to have prospective follow-up after their first visit, but they were not required to receive their treatment at DFCI. Clinical, pathology, and treatment data were manually abstracted by chart review. Estrogen receptor (ER) status, HER2 IHC score, and HER2 ISH status were abstracted from pathology records. HER2 status was defined according to the American Society of Clinical Oncology/College of American Pathologists (ASCO/CAP) Guidelines used at the time of diagnosis. This study involved analysis of retrospective data from IBC patients treated at DFCI who had provided written informed consent for the collection and use of these data for research purposes under IRB-approved Dana-Farber/Harvard Cancer Center Protocols.

### Study endpoints

PFS was defined as the time from mIBC diagnosis to LRPR, distant progression/relapse, or death. In the absence of a PFS event, the endpoint was censored at the date of last follow-up. OS was defined as the time from mIBC diagnosis to death from any cause. In the absence of an OS event, the endpoint was censored at the date last known alive. For patients who underwent surgery on the primary tumor, pCR was defined as the absence of residual invasive carcinoma in the breast and axillary lymph nodes, and survival was also estimated from the date of surgery.

### Statistical analyses

The distributions of PFS and OS were estimated using the Kaplan-Meier method, summarized by median and reported with 95% CI. The cumulative incidence function of CNS metastasis after mIBC diagnosis (among those without CNS metastasis at mIBC diagnosis) was estimated with death as a competing risk. The cumulative incidence of LRPR was estimated using a landmark approach^20^, defined from 12 months post-mIBC diagnosis (among those patients alive and LRPR-free at 12 months) with death as a competing risk. The 12-month landmark was selected given that most patients who underwent mastectomy did so within 12 months of the mIBC diagnosis.

### Reporting summary

Further information on research design is available in the Nature Research Reporting Summary linked to this article.

## Supplementary information

Supplemental Material

Reporting Summary

## Data Availability

The data that support the findings of this study are available from the corresponding author upon reasonable request.
