# Supplementary material for: Clinical outcomes of de novo metastatic HER2-positive inflammatory breast cancer
Source: NPJ Breast Cancer. 2023 Jun 2;9:50. doi: 10.1038/s41523-023-00555-w (PMC10238481; doi:10.1038/s41523-023-00555-w)

SUPPLEMENTARY MATERIAL

Supplementary Tables: 2

Supplementary Figures: 1

Supplementary Table 1. Clinicopathologic characteristics of study cohort – *de novo* HER2-positive metastatic IBC. *Defined hierarchically; Visceral: lung, liver, peritoneal, pleural; bone +/- lymph node also includes contralateral breast/skin involvement for 2 patients. **Contralateral axillary and/or distant nodal disease. ***No chemotherapy: one patient who had T-DM1; one patient had HP and never received chemotherapy. HR, hormone receptor; HER2, human epidermal growth factor receptor 2; CNS, central nervous system; LN, lymph node; AC, doxorubicin + cyclophosphamide; T-DM1, trastuzumab emtansine; IBC, inflammatory breast cancer

**Clinicopathologic Characteristics**		**No Surgery**		**Surgery**		**Overall**	
		**N**	**%**	**N**	**%**	**N**	**%**
		37	100%	41	100%	78	100%
Median age at diagnosis, years (range)		57 (24-91)		52 (28-68)		53 (24-91)	
HR status	HR-negative	21	56.8%	16	39.0%	37	47.4%
	HR-positive	16	43.2%	25	61.0%	41	52.6%
Number of metastatic sites at presentation (range: 1-7)	1	12	32.4%	30	73.2%	42	53.8%
	2	16	43.2%	7	17.1%	23	29.5%
	3	6	16.2%	3	7.3%	9	11.5%
	≥4	3	8.1%	1	2.4%	4	5.1%
Metastatic sites at presentation*	CNS +/- other	3	8.1%	1	2.4%	4	5.1%
	Visceral +/- other	21	56.8%	19	46.3%	40	51.3%
	Bone +/- lymph node	13	35.1%	21	51.2%	34	43.6%
Metastatic involvement at presentation	Bone	20	54.1%	17	41.5%	37	47.4%
	Lymph node**	19	51.4%	16	39.0%	35	44.9%
	Liver	12	32.4%	15	36.6%	27	34.6%
	Lung	12	32.4%	6	14.6%	18	23.1%
	CNS	3	8.1%	1	2.4%	4	5.1%
	Peritoneal/pleural	3	8.1%	-	-	3	3.8%
	Contralateral breast	2	5.4%	1	2.4%	3	3.8%
	Soft tissue	1	2.7%	-	-	1	1.3%
	Adrenal gland	1	2.7%	-	-	1	1.3%
	Bone marrow	1	2.7%	-	-	1	1.3%
Initial HER2-directed therapy	H/Pertuzumab (HP)	22	59.5%	18	43.9%	40	51.3%
	Trastuzumab (H)	14	37.8%	23	56.1%	37	47.4%
	T-DM1	1	2.7%	-	-	1	1.3%
Chemotherapy with initial HER2-directed therapy	Taxane	21	56.8%	21	51.2%	42	53.8%
	Taxane + platinum	3	8.1%	9	22.0%	12	15.4%
	Vinorelbine	8	21.6%	3	7.3%	11	14.1%
	AC + taxane	1	2.7%	7	17.1%	8	10.3%
	No chemotherapy***	2	5.4%	-	-	2	2.6%
	Taxane + capecitabine	1	2.7%	-	-	1	1.3%
	Taxane + cyclophosphamide	-	-	1	2.4%	1	1.3%
	Taxane + vinorelbine	1	2.7%	-	-	1	1.3%

Supplementary Table 2. Treatment details in patients with *de novo* HER2-positive metastatic IBC (mIBC) who underwent surgery (n=41/78). IBC, inflammatory breast cancer; HER2, human epidermal growth factor receptor 2; RCB, residual cancer burden; RT, radiation therapy; ALND, axillary lymph node dissection; SLNB, sentinel lymph node biopsy; pCR, pathologic complete response

**Treatment Details**		**Overall**	
		**N**	**%**
N patients		41	100%
Median time from mIBC diagnosis to surgery, months (range)		7.5 (4.2-46.9)	
Pre-operative HER2-directed therapy	Trastuzumab	20	48.8%
	Trastuzumab/pertuzumab	18	43.9%
	None (chemotherapy alone)	3	7.3%
Surgical procedure	Mastectomy + ALND	36	87.8%
	Mastectomy + SLNB	1	2.4%
	Mastectomy + unknown nodal sampling	4	9.8%
Number of lymph nodes removed	0 (no sampling of ipsilateral axilla)	4	9.8%
	1-3	1	2.4%
	4-9	20	48.8%
	≥10	15	39.0%
	Unknown	1	2.4%
Residual invasive disease in breast	Yes	26	63.4%
	No	14	34.1%
	Unknown	1	2.4%
Number of positive lymph nodes	- (no sampling)	4	9.8%
	0	17	41.5%
	1-3	11	26.8%
	≥4	9	22.0%
Pathologic response	pCR (RCB 0)	10	24.4%
	Residual disease	29	70.7%
	RCB-I	4	9.8%
	RCB-II	6	14.6%
	RCB-III	2	4.9%
	RCB class unknown	17	41.5%
	Unknown	2	4.9%
Radiation therapy for locoregional disease	No	8	19.5%
	Yes	33	80.5%
	Pre-operative	3	7.3%
	Post-operative RT	30	73.2%

Supplementary Figure 1. Cumulative incidence of CNS metastasis, with competing risk of death, among patients with de novo HER2-positive metastatic inflammatory breast cancer (mIBC). Among patients without known CNS metastasis at diagnosis of de novo HER2-positive mIBC (n=74), the cumulative incidence of CNS metastasis was 15.8% (95% CI: 8.3-25.4) and 23.8% (95% CI: 14.3-34.7) at 1 and 2 years, respectively. Cumulative incidence of CNS metastasis is shown as a blue line; competing risk of death is shown as a dashed black line. CI, confidence interval; CNS, central nervous system; HER2, human epidermal growth factor receptor 2; yr, year

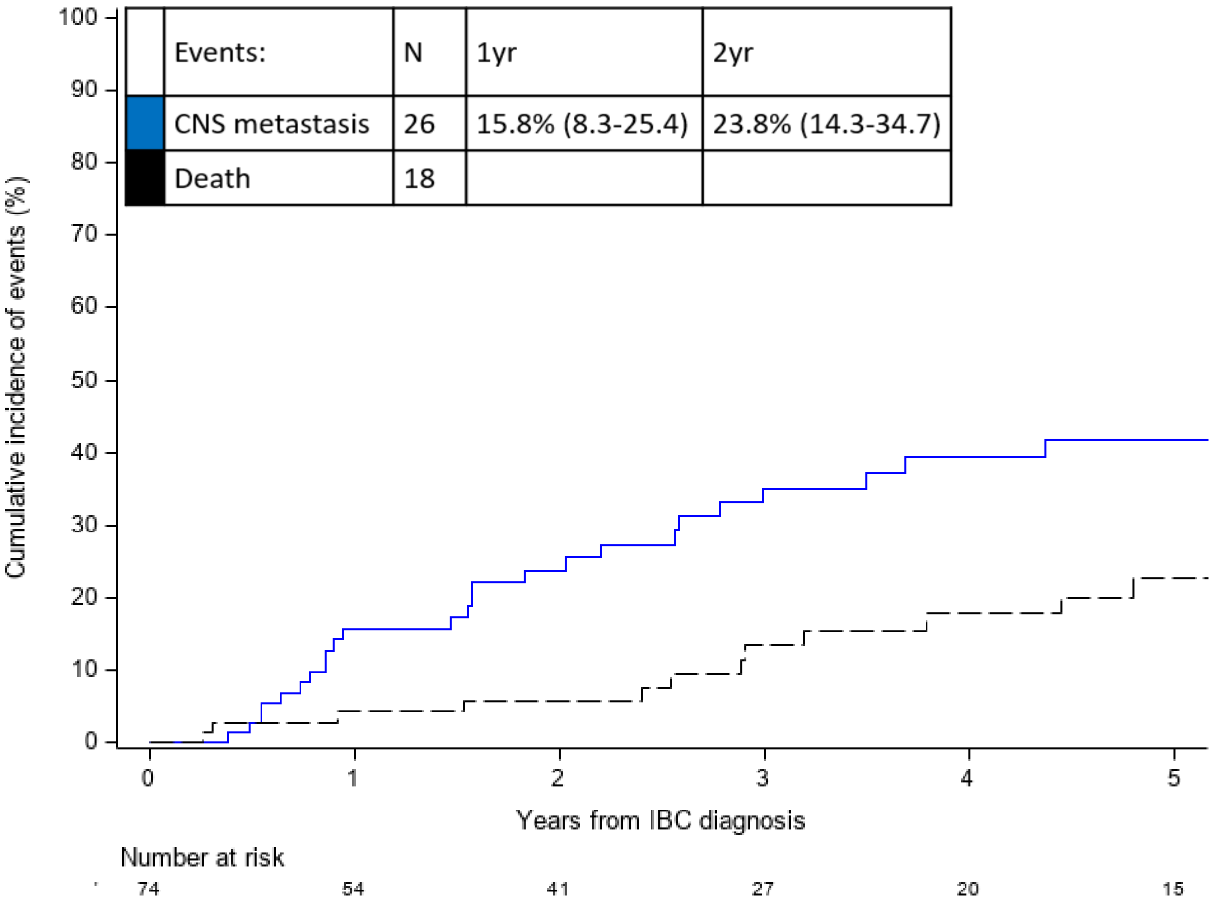

Supplement: Supplementary file 1 — Supplemental Material [file 41523_2023_555_MOESM1_ESM.pdf]
