# Supplementary material for: Clinical outcomes of de novo metastatic HER2-positive inflammatory breast cancer
Source: NPJ Breast Cancer. 2023 Jun 2;9:50. doi: 10.1038/s41523-023-00555-w (PMC10238481; doi:10.1038/s41523-023-00555-w)
Supplement: Supplementary file 2 — Reporting Summary [file 41523_2023_555_MOESM2_ESM.pdf]

Corresponding author(s): Filipa Lynce
Last updated by author(s): May 18, 2023

Reporting Summary

Nature Portfolio wishes to improve the reproducibility of the work that we publish. This form provides structure for consistency and transparency in reporting. For further information on Nature Portfolio policies, see our Editorial Policies and the Editorial Policy Checklist.

Statistics

For all statistical analyses, confirm that the following items are present in the figure legend, table legend, main text, or Methods section.

n/a	Confirmed	
☐	☒	The exact sample size (*n*) for each experimental group/condition, given as a discrete number and unit of measurement
☐	☒	A statement on whether measurements were taken from distinct samples or whether the same sample was measured repeatedly
☐	☒	The statistical test(s) used AND whether they are one- or two-sided<br>*Only common tests should be described solely by name; describe more complex techniques in the Methods section.*
☐	☒	A description of all covariates tested
☐	☒	A description of any assumptions or corrections, such as tests of normality and adjustment for multiple comparisons
☐	☒	A full description of the statistical parameters including central tendency (e.g. means) or other basic estimates (e.g. regression coefficient) AND variation (e.g. standard deviation) or associated estimates of uncertainty (e.g. confidence intervals)
☒	☐	For null hypothesis testing, the test statistic (e.g. *F*, *t*, *r*) with confidence intervals, effect sizes, degrees of freedom and *P* value noted<br>*Give P values as exact values whenever suitable.*
☒	☐	For Bayesian analysis, information on the choice of priors and Markov chain Monte Carlo settings
☒	☐	For hierarchical and complex designs, identification of the appropriate level for tests and full reporting of outcomes
☒	☐	Estimates of effect sizes (e.g. Cohen's *d*, Pearson's *r*), indicating how they were calculated

Our web collection on statistics for biologists contains articles on many of the points above.

Software and code

Policy information about availability of computer code

Data collection	Retrospective chart review. Data collected in RedCap.
Data analysis	N/A

For manuscripts utilizing custom algorithms or software that are central to the research but not yet described in published literature, software must be made available to editors and reviewers. We strongly encourage code deposition in a community repository (e.g. GitHub). See the Nature Portfolio guidelines for submitting code & software for further information.

Data

Policy information about availability of data

All manuscripts must include a data availability statement. This statement should provide the following information, where applicable:

- Accession codes, unique identifiers, or web links for publicly available datasets
- A description of any restrictions on data availability
- For clinical datasets or third party data, please ensure that the statement adheres to our policy

The data that support the findings of this study are available from the corresponding author upon reasonable request.

Human research participants

Policy information about studies involving human research participants and Sex and Gender in Research.

Reporting on sex and gender	All patients were of female sex.
Population characteristics	The median age at diagnosis was 53 years (range 24-91 years). Details of treatments received are included on Table 1.
Recruitment	Patients were all seen at the Dana Farber Inflammatory Breast Cancer Program and were offered participation in this registry. To be eligible for inclusion in this study, patients were required to have prospective follow-up after their first visit, but they were not required to receive their treatment at DFCI.
Ethics oversight	Dana-Farber IRB

Note that full information on the approval of the study protocol must also be provided in the manuscript.

Field-specific reporting

Please select the one below that is the best fit for your research. If you are not sure, read the appropriate sections before making your selection.

☒ Life sciences ☐ Behavioural & social sciences ☐ Ecological, evolutionary & environmental sciences

For a reference copy of the document with all sections, see nature.com/documents/nr-reporting-summary-flat.pdf

Life sciences study design

All studies must disclose on these points even when the disclosure is negative.

Sample size	Convenience sample size - all patients who signed consent to participate in our registry.
Data exclusions	N/A
Replication	N/A
Randomization	N/A
Blinding	N/A

Reporting for specific materials, systems and methods

We require information from authors about some types of materials, experimental systems and methods used in many studies. Here, indicate whether each material, system or method listed is relevant to your study. If you are not sure if a list item applies to your research, read the appropriate section before selecting a response.

Materials & experimental systems

n/a	Involved in the study	
☒	☐	Antibodies
☒	☐	Eukaryotic cell lines
☒	☐	Palaeontology and archaeology
☒	☐	Animals and other organisms
☐	☒	Clinical data
☒	☐	Dual use research of concern

Methods

n/a	Involved in the study	
☒	☐	ChIP-seq
☒	☐	Flow cytometry
☒	☐	MRI-based neuroimaging

Clinical data

Policy information about clinical studies

All manuscripts should comply with the ICMJE guidelines for publication of clinical research and a completed CONSORT checklist must be included with all submissions.

Clinical trial registration	N/A - not a clinical trial

March 2021

Study protocol	N/A
Data collection	Patients diagnosed with de novo HER2+ mIBC between 1998-2019 were identified from an IRB-approved IBC registry at Dana-Farber Cancer Institute (DFCI).
Outcomes	The primary endpoint was PFS. PFS was defined as the time from mIBC diagnosis to LRPR, distant progression/relapse, or death. In the absence of a PFS event, the endpoint was censored at the date of last follow-up. OS was defined as the time from mIBC diagnosis to death from any cause. In the absence of an OS event, the endpoint was censored at the date last known alive. For patients who underwent surgery of the primary tumor, pCR was defined as the absence of residual invasive carcinoma in the breast and axillary lymph nodes, and survival was also estimated from the date of surgery.
